# Independent control of COVID-19 vaccines by EU Official Control Authority Batch Release: challenges, strengths and successes

**DOI:** 10.1038/s41541-023-00617-x

**Published:** 2023-02-23

**Authors:** Catherine Milne, Ralf Wagner, François Cano, Martijn Bruysters, Geneviève Waeterloos, Dieter Pullirsch, Michael Wierer, Laurent Mallet

**Affiliations:** 1 European Directorate for the Quality of Medicines & HealthCare, Department of Biological Standardisation, OMCL Network and HealthCare, Council of Europe, Strasbourg, France; 2 Paul-Ehrlich-Institut, Federal Institute for Vaccines and Biomedicines, Langen, Germany; 3 French National Agency for Medicines and Health Product Safety (ANSM), Laboratory Controls Division, Lyon, France; 4 Dutch National Institute for Public Health and Environment, Centre for Health Protection (GZB), Bilthoven, The Netherlands; 5 Sciensano, Scientific Directorate Biological Health Risks, Service quality of vaccines and blood products, Brussels, Belgium; 6 Austrian Federal Office for Safety in Health Care, Austrian Agency for Health and Food Safety, Vienna, Austria

**Keywords:** RNA vaccines, Drug regulation, Viral infection, Protein vaccines, Inactivated vaccines

## Abstract

Vaccines have been a key tool in stemming the tide of the COVID-19 pandemic. The rapid development of effective vaccines against COVID-19, together with their regulatory approval and wide scale distribution has been achieved in an impressively short period thanks to the intense efforts of many. In parallel to vaccine development, the EU considered it important to prepare for the independent control of the COVID-19 vaccines, including testing, to help ensure that only vaccines that comply with the approved quality requirements reach the public and to help improve/increase public confidence in the vaccines. The existing EU Official Control Authority Batch Release (OCABR) system, co-ordinated by the European Directorate for the Quality of Medicines and HealthCare (EDQM), was able to effectively respond to the need, through rapid co-ordination, work-sharing, advance planning and early interaction with manufacturers, the Coalition for Epidemic Preparedness Innovation (CEPI) and regulatory authorities. The Official Medicines Control Laboratories (OMCLs) involved in the OCABR activity, using the strength of the established system in the OCABR network and adaptations to the crisis conditions, were ready to release the first COVID-19 vaccine batches, after protocol review and testing, at the time of the conditional marketing authorisation for each of the COVID-19 vaccines, with no delay for batches reaching the public. Thanks to the dedication of resources by the EU and national authorities as well as by the EDQM, this was done without impacting the release of the other vaccines and human blood and plasma derived medicinal products, essential for public health. Transparency and communication of practices were important factors to support reliance on the OCABR outcome in non-EU countries, with the goal to improve access to vaccines in Europe and beyond. An overview of the process, legal background, challenges and successes of OCABR for COVID-19 vaccines as well as a look at the international perspective and lessons learned is provided.

## Vaccines in response to the COVID-19 Pandemic

Since its first emergence in Wuhan/China in December 2019, the coronavirus disease 2019 (COVID-19) caused by the severe acute respiratory syndrome coronavirus 2 (SARS-CoV-2) very rapidly spread across the globe to cause a pandemic. The virus readily transmits from human to human thus giving rise to high infection rates in the affected population. In many cases, SARS-CoV-2 infection either remains asymptomatic or causes only mild respiratory or flu-like symptoms such as fever, chills, cough, fatigue, headache, nausea and/or vomiting^1^. However, especially in elderly or immune-compromised persons the disease may also progress into severe COVID-19 with pneumonia and acute respiratory distress syndrome requiring hospitalisation or even leading to death. Until end of July 2022, globally more than 570 million cases of COVID-19 (in accordance with the applied case definitions and testing strategies in the affected countries) have been reported, including over 6 million fatal outcomes^2^. These numbers highlight the enormous and devastating impact the pandemic has on public health and the huge socio-economic consequences attached to this global crisis.

Personal protection, specific hygiene concepts and public health countermeasures can be applied to reduce the infection rates. However, amidst the growing pandemic safe and effective prophylactic SARS-CoV-2 vaccines were seen as the prime and most urgently needed preventive countermeasure to protect the population from contracting the infection or developing COVID-19. There is no doubt that effective vaccines are an indispensable contribution to a permanent solution to stop the pandemic and to allow societies to return to normal life conditions. This urgent need immediately set off a tremendous global run for the discovery and development of COVID-19 vaccines and only weeks after the pandemic had been officially declared by the World Health Organisation (WHO) more than a hundred vaccine candidates had entered into preclinical or early clinical investigational phase^3^. The frontrunners among these multiple developmental strategies were those candidates representing platform technologies for which related vaccines against similar infectious agents had either been approved before or at least advanced into the clinical evaluation phase. Such vaccine candidates, namely those based on mRNA technology or using adenoviral vectors, proceeded into phase II and phase III clinical development with unprecedented velocity within only a few months. In order to keep pace with this highly accelerated vaccine development, regulatory authorities around the globe rapidly established COVID-19 specific standards for the conduct and the objectives of clinical trials and for vaccine licensure or emergency listing. These regulatory requirements were flexible enough to allow for the marked acceleration of vaccine development and licensing procedures but sufficiently rigorous and imperative to control and ensure the safety and efficacy of the vaccine candidates including the establishment of product quality controls and specifications and the assurance of Good Manufacturing Practices (GMP)^4^. For licensing procedures organised by the European Medicines Agency (EMA), acceleration was achieved mainly by drastically shortening the timelines for regulatory evaluation by allowing for data submission in the form of a “rolling review”, in which discrete data packages can be submitted for immediate regulatory review as soon as they become available, in advance of the formal request for marketing authorisation^5^.

In the absence of an immunological correlate of protection vaccine efficacy had to be demonstrated in huge clinical studies comprising 30,000–40,000 subjects investigating the candidate´s ability to protect against symptomatic COVID-19^6,7^. Safety monitoring also had to be compliant with the regulatory requirements as laid down for the regulatory evaluation and licensure of conventional vaccines^8^. Collection and analysis of these comprehensive and essential clinical data sets were completed in an unprecedentedly short period of only several months and revealed that the vaccines under development were highly efficacious and safe^9^. These tremendous joint efforts of all the involved stakeholders eventually led to the granting of conditional marketing authorisations for two mRNA-based vaccine in the European Union (EU) within less than a year^5^. Soon after, two adenoviral vector vaccines received conditional marketing authorisations with other vaccines using different platforms to follow later. Table 1 provides an overview of COVID-19 vaccines that have been granted a conditional marketing authorisation or regular marketing authorisation in the EU as of July 2022.

**Table 1:** Overview of COVID-19 vaccines licensed in the EU as of 01/07/2022 with conditional authorisation and first release dates.

Vaccine name	Company	Platform	EU conditional authorisation date	First protocol submitted	First OCABR release
Comirnaty	BioNTech/Pfizer	mRNA	21/12/2020^c^	21/12/2020	22/12/2020
Spikevax	Moderna	mRNA	06/01/2021^b^	06/01/2021	06/01/2021
Vaxzevria	AstraZeneca	Adenoviral vector	29/01/2021	01/02/2021	01/02/2021
JCovden	Janssen	Adenoviral vector	11/03/2021	08/04/2021	09/04/2021
Nuvaxovid	Novavax	Recombinant protein + adjuvant	20/12/2021	20/02/2022	21/02/2022
COVID-19 Vaccine (inactivated, adjuvanted)	Valneva	Inactivated, adjuvanted	24/06/2022^a^	12/08/2022	16/08/2022

^a^regular authorisation initially, ^b^regular authorisation approved 03/10/2022, ^c^regular authorisation approved 10/10/2022.

The COVID-19 vaccines and candidates are very diverse in nature and technology (mRNA, viral vector, recombinant protein, inactivated viral vaccine, with certain vaccines using adjuvants, some new to vaccine formulations). For some platforms e.g., mRNA, and to a lesser extent viral vector vaccines, only limited, if any, experience existed regarding their manufacturing processes and inherent control strategies at the time of regulatory evaluation for licensure. Furthermore, some of the licensed vaccines are from manufacturers that have only recent expertise in the field of human vaccine production. Stringent control of the intended quality and batch-to-batch consistency of every COVID-19 vaccine was paramount, considering any given batch could be applied to millions of people during the mass vaccination campaigns in the EU. The rapid development and authorisation of this variety of new vaccines against COVID-19 therefore required also a rapid preparation for independent control of the vaccines.

Indeed, in the EU, for most member states it is required that vaccine batches (also referred to as lots) are experimentally tested for key quality-assuring properties by an independent laboratory, called an Official Medicines Control Laboratory (OMCL) in a procedure called Official Control Authority Batch Release (OCABR)^10^. Based on the elements noted above, efficient independent control of COVID-19 vaccines was considered a priority. Independent control of COVID-19 vaccines, to support public health and contribute to public confidence in these vaccines developed during the crisis, is also recognised as important by countries outside the EU^11,12^.

Hence, in order not to delay access to vaccines following their marketing authorisation, OMCLs had to get ready to exert adequate and comprehensive control of COVID-19 vaccines and all preparatory work and provisions had to be tackled and completed in parallel to the accelerated licensing procedure. The primary goal was to have the OMCL testing for COVID-19 vaccines fully established by the time of licensure.

## Official Control Authority Batch Release

The EU Official Control Authority Batch Release (OCABR) procedure is foreseen in EU legislation, in article 114 of the Directive 2001/83/EC as amended^13^. If this EU legislation is introduced in national legislation (which for vaccines is the case for the majority of EU member states), it enforces that each batch of vaccine, including vaccines against COVID-19, is to be controlled by an independent national laboratory, an OMCL, before it reaches the public. Importantly, article 114 also requires mutual recognition of results amongst member states in order to maximise efficiency and avoid unnecessary repeat testing or delay of product to market.

OCABR is thus an additional guarantee of the quality of these sensitive products. Its use is based on an underlying risk assessment that recognises the fact that vaccines are biologicals and have the potential for inherent variability due their production and testing systems. This procedure also applies to medicinal products derived from human blood or plasma (e.g., clotting factor, immunoglobulin and albumin) which are not discussed further here.

The elements of the EU OCABR system are in line with the WHO guidance for vaccine lot release, which recommends independent control of every vaccine lot through protocol review and relevant testing^14^.

The OMCLs that carry out OCABR are part of the General European OMCL Network (GEON). Established in 1994 the network is co-sponsored by the Council of Europe (COE) and the European Commission. The European Directorate for the Quality of Medicines and HealthCare (EDQM), of the COE is the network secretariat^15^. The OCABR network members are a subset of the GEON that includes only EU/European Economic Area (EEA) member states as well as countries having signed an official agreement with the EU, such as Switzerland and Israel^16^.

All of the member states are legally bound to recognise the test results from OCABR. The network has many advantages, including work-sharing, synergy, exchange of expertise and experience as well as providing a common voice for interactions with external partners like manufacturers.

**Fig. 1:**
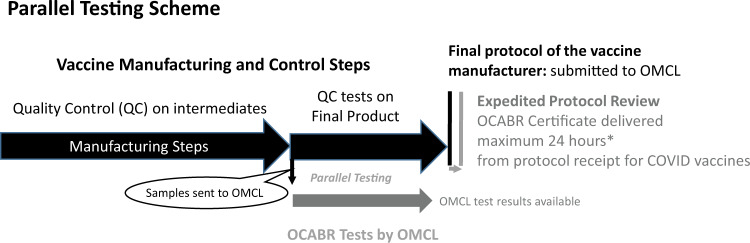
Principle of parallel testing during OCABR. Black horizontal arrows denote elements in the manufacturer’s production and control process; grey horizontal arrows represent elements of the OCABR process at the OMCL. Black vertical line is release of the manufacturer’s protocol with QC results to the OMCL, grey vertical line is the release of the OCABR certificate for compliant batches *Represents the timing in the first months of the COVID-19 campaigns, subsequently the average range was 1–3 days.

For OCABR, each batch of vaccine is controlled by an OMCL by testing a specific subset of the tests from the approved marketing authorisation dossier, as agreed by all member states. The choice of tests is based on added value for the independent review. The OMCL also reviews the manufacturers test results, which are included in a batch protocol, which is part of the OCABR product specific guidelines^17^. If the data in the manufacturer’s protocol and the results from the tests performed at the OMCL are compliant with the approved specifications in the marketing authorisation, the batch is granted an EU OCABR certificate, which is given to the manufacturer. The manufacturer can use the OCABR certificate when applying to place the batch on the market in any of the network member countries. If a batch is not compliant, it does not receive a certificate and all members and recognised observers are informed of the status through an internal information system between competent authorities. The legislation^13^ denotes 60 days to carry out this procedure. However, under normal conditions, OMCLs provide OCABR certificates in the range of 1–7 working days from the time of receipt of the completed protocol from the manufacturer. The time may be longer in some cases, for example if there is the need to repeat testing, an investigation is ongoing or if the manufacturer did not use the opportunity of parallel testing as described in Fig. 1.

To avoid duplication of work any given batch should be tested in only one OMCL, however to ensure business continuity and improve networking it is recommended that more than one OMCL is prepared to test any vaccine.

It is the responsibility of the manufacturer to arrange with OMCLs for OCABR well in advance of the expected release of the first batch. This should take place ahead of the actual marketing authorisation application, at a point when the control strategy proposal is defined, test methods have been validated and there is a clear plan for the timing of the application for a marketing authorisation based on promising indications of vaccine safety and efficacy from clinical trials. The OMCLs must prepare for batch release by transferring and verifying the relevant methods, in accordance with the list of tests chosen by the OCABR Network. This takes place after the agreement with the manufacturer and continues during the marketing authorisation approval process. In the EU the centralised authorisation procedure is a codified process, formally taking 210 active days from the time of validation of the applications^18^ but in real time, including clock stops that allow the applicant to respond to questions raised by assessors, it can take at least a year. As such, the total time for normal technical transfer can take between a year to 18 months without causing any delay to the market. Over the whole history of the OCABR network, by applying these practices, OMCLs have always been ready at the time of release requests for the first approved vaccine batches. While the manufacturer may choose the OMCL, some conditions apply and the OMCL must be externally audited and comply with ISO/IEC 17025 quality requirements and have the competence and capacity for testing. They also must agree to carry out the OCABR. OMCLs are by definition independent from the manufacturer.

A key concern of the OMCLs is to carry out OCABR without delaying the vaccine batches from reaching the recipients. This is managed primarily by use of parallel testing (Fig. 1). This means that as soon as the manufacturer begins their own quality control tests, they send samples to the OMCL who does the independent testing in parallel. The OMCL will then have completed its tests by the time the manufacturer sends their test results in the protocol. The OMCL has then only to do the critical paper review of the protocol before providing the decision in a short time frame.

**Fig. 2:**
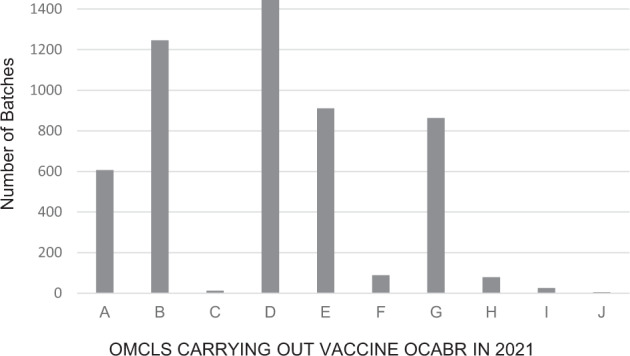
Number of vaccine batches undergoing OCABR at each OMCL in 2021. OMCLs are represented anonymously by letter on the X-axis. The Y-axis represents number of batches, all vaccine types combined.

The network system provides opportunities for work sharing, strengthening competence and common problem solving. In 2021, eleven member OMCLs participated in OCABR of vaccines, contributing according to their capacity and expertise. While COVID-19 vaccines have been a key focus since the end of 2020, all other vaccines essential for public health were also released by the OMCLs to the order of 3600 batches of non-COVID-19 vaccines on top of the more than 1600 COVID-19 vaccine batches released in 2021 (Fig. 2). All of these vaccines were then accessible for use within the full EU/EEA via mutual recognition of the OCABR certificate.

The value of the OCABR certificate does not stop at EU/EEA borders. The EU OCABR network has signed memoranda of understanding (MOU) for vaccines with several non-EU/EEA authorities such as Health Canada, the Therapeutic Goods Administration, Australia and the National Institute for Biological Standards and Control, UK. These partners have the status of OCABR network observer. The MOU allows for confidential exchange of information on batch release activity but does not include mandatory mutual recognition. It does however promote exchange of good practice and promote reliance. Numerous other countries outside the EU/EEA rely on the OCABR certificates. Many request them as part of their own release process and ideally, this prevents re-testing of the same batch in those countries, which means that doses are not wasted and the public receives these important medicines more quickly.

## OCABR for COVID-19 vaccines

Already at the end of 2019 and in early 2020 there were growing signals that a health crisis of global scope was building and the network followed the situation closely. Concrete emergency planning began in earnest when signals were received from the European Commission and EU national authorities that an EU wide plan to deal with COVID-19 was being developed that involved accelerated authorisation of COVID-19 vaccines.

In the context of the pandemic, an accelerated preparation of the key elements for OCABR was thus also needed. This preparation was focused on two axes:

- The preparation of the OCABR guidelines in parallel with the dossier evaluation by the EMA for the first vaccines (mRNA based and viral-vectored based) which were anticipated to obtain a conditional marketing authorisation.
- The early transfer of the selected analytical methods to the OMCLs in order to ensure the readiness of the OMCLs and EDQM when the first batches were available and the first conditional marketing authorisations were delivered.

Starting in March 2020, the EDQM mobilised the EU OCABR Network members and facilitated an early exchange with manufacturers. This mobilisation was efficient due to the commitment of national authorities and of the OMCLs to prioritise these key batch release activities and to dedicate resources with, in some cases, an increase in the workforce. Preparedness required anticipation of all the steps to ensure the readiness, as well as a retro planning i.e., planning from the deadline backwards, based on the assumptions that were available. The anticipation of the different steps was however associated with the risk that some products may not be accepted by the EMA.

The early exchanges with manufacturers was facilitated by a close collaboration with the Coalition for Epidemic Preparedness Innovations (CEPI)/COVAX and the willingness of the manufacturers to share their analytical strategies for their vaccine candidates in early phases of their development.

In addition, continuous communication with the European Commission and the EMA was critical to ensure an alignment of the batch release preparation with the timing of the conditional marketing authorisations.

The mobilisation of the OCABR Network led to the drafting of a recommendation document for manufacturers on early method transfer and an OMCL competency list, based on control techniques for the different categories of COVID-19 vaccine candidates, to help the manufacturers identify OMCLs with the relevant skill sets. Both documents were provided to known manufacturers directly and through the manufacturers’ association Vaccines Europe in July 2020 and were subsequently supplied to COVID-19 manufacturers on request throughout 2021 and 2022. The competency list was updated twice in 2021 to keep up to date with the evolving situation.

Early dialogue with the manufacturers through CEPI, industry associations and dedicated meetings with individual manufacturers allowed the exchange and collection of confidential information related to the different vaccine candidates necessary to prepare the OCABR guidelines. This early dialogue was also an opportunity to promote the following best practices:

- An early transfer of the methods by the manufacturers to the OMCL(s) while these methods had completed proof of concept regarding their suitability for the intended use but still had to complete full validation.
- The selection of more than one OMCL by each manufacturer to secure the probability of having one OMCL ready for the first batches and to be able to anticipate the large number of batches which were announced for each vaccine.
- The importance of developing in vitro tests (e.g., for potency assays) at the level of the manufacturers, as part of our engagement on 3Rs principles and to facilitate the transfer of the methods to the OMCLs.

Since new companies/manufacturers, which were less familiar with the batch release process in Europe, were also among the front-runners, this early dialogue was also important to provide them with a clear explanation of the OCABR process. It was particularly critical for the early selection of the OMCLs for their candidate vaccines and for further technical exchanges and transfer of the selected analytical methods to the OMCLs. Regular joint meetings between the selected OMCLs and the individual manufacturers were subsequently organised to facilitate the technical and logistical discussions between the concerned stakeholders.

In November 2020, the EDQM published three new OCABR guidelines outlining the tests to be performed by OMCLs in the EU OCABR Network as part of the independent control of the first anticipated pandemic COVID-19 vaccines. This was unprecedented since at the time, no COVID-19 vaccines had yet received an EU conditional marketing authorisation. However, these guidelines were made available at an early stage for transparency, to help anticipate the launch of the first vaccines and to allow OMCLs and manufacturers to take the necessary steps to prepare for OCABR, thus preventing delays in availability while still ensuring their quality and safety. The publication of the guidelines at this early stage was also done with the goal to allow global authorities access to the OCABR testing strategy to promote common practice and/or reliance. This process continued for other COVID-19 vaccines in the pipeline, such as inactivated vaccines and others (Table 2).

**Table 2:** List of tests to be carried out by OMCLs as described in section 2 of OCABR guidelines for COVID-19 Vaccines*.

OMCL tests in OCABR guidelines for pandemic COVID-19 vaccines	
Non-replicating adenovirus vectored vaccine	Tests on the final lot: • Appearance • Identity (potency may serve as an identity test) • Potency
mRNA vaccine	Tests on the final lot: • Appearance • Identity • Potency • Integrity
Recombinant spike protein vaccine	Test on the bulk: • Purity (depending on the MA, if not performed on the final lot) Tests on the final lot: • Appearance • Identity (potency may serve as an identity test) • Potency • Purity (depending on the MA)
Inactivated vaccine	Tests on the final lot: • Appearance • Identity (potency may serve as an identity test) • Potency

MA refers to the approved conditional or regular marketing authorisation

*Tests listed as published in July 2022; guidelines may be subject to revision and the latest versions can be accessed on the EDQM website www.edqm.eu.

This crucial achievement was made possible through dialogue and co-operation between the EDQM, OMCLs and manufacturers. All these initiatives meant that the EDQM and the OCABR Network were ready when the first COVID-19 vaccines were given a conditional marketing authorisation in December 2020 and January 2021. This also meant that they were able to ensure that vaccine doses were available for the launch of vaccination in the EU member states from the end of 2020.

## What were the challenges?

Implementation of the OCABR batch release of any new product poses challenges for an OMCL, including the need for technology transfer/validation, staff training, drafting of new quality documents, etc.

The release of COVID-19 vaccines was even more challenging due to specific additional factors:

### Regulatory context

For the COVID-19 vaccines, the conditional marketing authorisation assessment included a rolling review and followed an accelerated process^19^ leading to a shorter time between the first contact/discussion with the manufacturer and the release of the first batches. For COVID-19 vaccines, this period between first contact and release was reduced from the usual 12 to 18 months to only a few months and in one case, preparation was finalised in only 4 weeks.

For some vaccines, the techniques used for the batch release tests evolved during the course of the dossier assessment and some specifications were changed just hours before the final granting of the conditional marketing authorisation thus requiring last minute adjustments for the first released batches.

### Investment risk

In the race to develop a vaccine there were numerous candidates around the globe using different technological approaches^3^. With no clear indication at the beginning which would be successful and which would arrive first, OMCLs and their member state authorities were obliged to take risks in investing in the preparation of OCABR for vaccines that may or may not reach the market. This required a commitment from the national governments and, at the EU level, a good co-operation throughout the network to spread the risk a much as possible. It also required a need to continuously monitor development at the regulatory level to stay in stride with the most advanced candidates.

### Multiple production and quality control sites

In order to be able to produce and control a large number of doses of vaccine in a short time frame, the manufacturers have multiplied the number of production and quality control sites for COVID-19 vaccines, including increased use of contract plants for manufacturing as well as contract testing facilities. From an OMCL perspective this aspect has meant dealing with a high number of different interlocutors, managing large amounts of information and maintaining a close follow up on the approval of the different sites in collaboration with EMA. For new production sites, it also required establishment of multiple shipment “verification routes” to secure the transport and receipt of the samples and necessary reagents.

### New products and controls

For COVID-19, new types of vaccines were developed and new methods for vaccine control at the OMCLs were needed, leading to the transfer of new methods not already used in the context of batch release. In many cases the purchase of new equipment was needed, which also entailed qualification, training and validation, once again in a short period of time. However, thanks to the agility and technical competence of the OMCLs, all the technical transfers were performed in the required time frame.

### Epidemic context

Of course, the epidemic context was an additional challenge for OMCLs with the potential to impact different aspects of the batch release process. The first anticipated effect was the lack of availability of staff, due to sickness or other reasons like travel restrictions. The OMCLs mitigated this risk by allocating the human resources available to the prioritised batch release process and putting on hold other less essential activities. Hiring of additional staff was also an option when this was feasible. Moreover teleworking was developed for some staff when applicable and in many cases staff worked in team rotations to prevent the spread of the virus within the OMCLs.

Another hurdle that arose was the possible shortage of some “basic” lab reagents (e.g., plates, tips, cell growth medium, columns) due to high demand all over the world. OMCLs were required in some instances to search for alternative vendors or reagents thus increasing the complexity and adding pressure on the process. On the other hand, and fortunately, the transport system for the shipment of reagents or samples from the manufacturer was not really impacted by the epidemic context.

At the network level a contingency plan was also developed to react in case of the inability to carry out OCABR in one or the other OMCL. Thanks to the careful planning and management at the individual OMCLs the plan never needed to be implemented.

### High demand and expectations

From a public health perspective, the availability of COVID-19 vaccines was greatly awaited, adding additional pressure. In consequence, the OMCLs were mobilised to guarantee the availability of high quality vaccines within days, and sometimes hours of receipt of the manufacturer’s completed protocols and thus, far below the official deadline of 60 days noted in the EU Directive 2001/83/EC as amended^13^. This required intensified work schedules with 7 days per week activity. It is also important to keep in mind that all the other vaccine and blood derived medicinal products included in the EU batch release system continued to undergo OCABR during the same period and the timing and number of lots released for those products were not affected by the COVID-19 crisis. To achieve all these challenges, the collaboration within the OCABR network was a paramount element: allowing the sharing of the workload between different OMCLs and exchange of information (regulatory and technical) easily and rapidly. Once again, the EU OMCL network has shown its resilience even during tense times. The importance of the EU batch release process has been recognised also outside the EU by international organisations (CEPI/COVAX, WHO, …) and by countries outside EU contacting EU OMCLs directly to have specific information on COVID-19 vaccines.

## What were the successes?

Despite the challenges the OMCLs of the network, working together and in co-operation with the regulators at EMA, the European Commission, the individual manufacturers, the manufacturer’s associations and CEPI/COVAX, have succeeded in the goal to independently evaluate batches of COVID-19 vaccines before they reach the public without delaying access to these critical medicines against the pandemic.

Indeed for each of the vaccines that received conditional marketing authorisation, the first batches were released by the OMCL with an EU OCABR certificate within days of the authorisation and receipt of the final protocol from the manufacturer (Table 1). This was the result of intensive upstream activity in the preparation of relevant guidance (publicly available product specific guidelines, method transfer guidance and OMCL competency list), transfer of necessary test methods to the OMCLs as described above and testing of batches in advance of approval in anticipation of application of the approved specifications. It demonstrated the scientific expertise and operational flexibility of the OMCLs who were able to implement new methods to the status of full transfer validation within tight timelines. It also clearly demonstrated that the European OMCL network is well prepared to cope with a pandemic situation in a timely manner. The achievement is also thanks to the commitment and rigour of both the OMCLS to attain this common goal and the involved manufacturers to interact with the OMCLs in a positive and proactive manner.

**Fig. 3:**
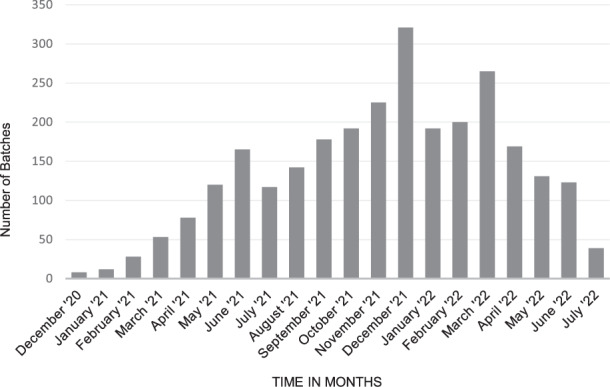
Overview of the number of COVID-19 vaccines batches released by EU OMCLs from December 2020 to July 2022. All authorised vaccine types combined. X-axis represents months from December 2020 to July 2022, Y-axis is the number of vaccine batches.

Throughout the release activity, thanks to efficient interaction with the manufacturers and the instrumental use of parallel testing, the issuing of OCABR certificates caused no delay for the vaccine batches reaching the public. From the release of the first batches of vaccine in December 2020 until the end of July 2022, OMCLs had carried out OCABR on more than 2750 batches of COVID-19 vaccines, representing billions of doses from all of the EU approved COVID-19 vaccines combined (Fig. 3). EU OCABR certificates issued for compliant batches provided an assurance of quality for vaccine recipients in the EU/EEA and beyond.

The goal of the independent testing and review of each batch pre-release is to prevent non-compliant batches reaching the vaccine recipients and to monitor the control systems of the manufacturer. In this respect, the OCABR system was highly successful. During the independent review and testing process a small number of batches were rejected, either during the testing or based on the protocol review. For example, during potency testing at an OMCL on one of the early vaccine batches, homogeneity problems between the filled vials was found, with a potency value below the specification for some vials within the same batch. Based on these OMCL results, an investigation ensued with the manufacturer. Following a regulatory decision, part of the batch was rejected and the analytical procedure at the level of the manufacturer was improved and included in the marketing authorisation. The number of batches rejected for quality reasons, while small, remains important since one batch represents millions of doses. This observation is similar to that for non-COVID-19 vaccines. Indeed, it is part of the normal manufacturing landscape for biological medicines to expect a small number of batches that are not compliant with the strict requirements due, for example, to biological variability and thus be rejected before reaching the market. The independent review batch-to-batch is an important reassurance for the public that only compliant batches are released to the vaccine recipients.

With an anticipated high demand for a large number of batches to serve the population of Europe and beyond the need to involve more than one OMCL per product was essential, both to manage the volume but also to consider business continuity. This provided several advantages, including a combined support, common meetings with the involved manufacturer and the ability to spread the risk to ensure at least one OMCL was prepared at the time of approval with the other(s) to complete the preparations once approval was granted.

Successes also included the increased use of videoconference platforms to meet efficiently in large or small groups, sometimes on very short notice, to take critical decisions at the network level, resolve problems or exchange important information. OMCLs also relied heavily on electronic submission of documentation from the manufacturers.

The EU OMCL network has a long-standing commitment to the application of the 3Rs (reduction, refinement, replacement of the use of animals) in the context of quality testing. The tests selected for OCABR were all in vitro methods. This was possible largely due to the commitment of the manufacturers and regulators to promote and validate these approaches. It was also a key factor for rapid release since tests involving animals are usually long (around 60 days) as compared to the in vitro alternatives (within hours to a week).

The full exploitation of the existing OCABR system and network can also be considered as a success. The established channels of communication, organisation of working groups, links between OMCLs and external partners, the excellent working relationship established and the co-ordination offered by the EDQM secretariat were possible thanks to the years of investment, development, and maintenance of this system.

The number of batches of COVID-19 vaccines released by EU OMCLS represents a number of doses far beyond the needs within EU/EEA. Indeed, more than half of all batches released by EU OMCLs, were used outside the EU/EEA with export to more than 160 non-EU countries^20,21^. The transparency of the EU OCABR system allows others countries to rely on EU certificates where possible depending on their own national requirements, as promoted by CEPI/COVAX and WHO, thus preventing additional testing control, which would slow down access and potentially add stress to the already stretched system of vaccine distribution.

## International perspective

In the EU, the regulation and control of medicines is well established. The EMA is the key regulator for medicines (including vaccines) undergoing the centralised procedure^18,22^. Therefore, a marketing authorisation issued by the European Commission (after scientific evaluation and recommendation of the EMA) is applicable in all EU member states. Similarly, the OCABR certificates (see above) issued by individual member states are valid in all EU member states.

Globally, the World Heath Organisation (WHO) recommends an independent batch release by National Control Laboratories (NCLs) for vaccines, at least by the critical review and approval of the manufacturers' protocol or through recognition/reliance of the evaluation from another control authority^14,23^. The WHO strives towards international reliance among national regulatory authorities^24^. For marketing authorisation, the WHO has a system of prequalification in place for (among other products) vaccines, providing assurance for quality, safety and efficacy of these products^25^. Prequalification of vaccines is a prerequisite for procurement of vaccines by UNICEF, and other UN agencies and is a key tool for other authorities in need of support for regulatory evaluation.

With respect to the control of vaccines by national authorities, outside the EU/EEA there is no legal basis for recognition between countries and the global system for batch release has a less codified framework. Currently WHO has established a network of national control laboratories (WHO-National Control Laboratory Network for Biologicals; WHO-NNB^26^) to promote reliance on batch release by national authorities from the country that produces the vaccines through communication and exchange between members. The main reason for this is to facilitate access to vaccines in the global markets and avoid redundant testing. Testing of vaccines by national authorities provides additional assurance of the vaccine quality also in non-pandemic times. It also needs to be considered however, that testing of vaccines can take substantial time (individual tests may take several weeks) and since individual batches of vaccines are rarely sent to one country as a whole, one batch may be tested by different countries. Furthermore, due to the complexity of biological tests, batches may fail due to technical or methodological errors rather than the inherent quality, or lack thereof, of the batch. The latter is especially relevant if testing laboratories are lacking specific technology or experience. Promoting reliance is therefore beneficial. Most EU OMCLs actively involved in OCABR of vaccines are also full members of the WHO-NNB and thus contribute to the exchange of information and experience that is the basis for the network.

During preparation for and later actual batch release of COVID-19 vaccines, the EU OMCL network has substantially contributed to the WHO-NNB. Firstly, the product specific guidelines drafted by OMCL experts were published on the EDQM website^17^ and were actively shared with WHO. By doing so, the consensus of EU experts on which tests were considered to be most important to be carried out independently by a national control laboratory was shared with the rest of the world. Via these guidelines, the expected amount of detail in the batch documentation from the manufacturer was also disclosed. This information may be useful to other countries when setting up testing plans for other/similar vaccines and could provide part of a common basis for discussion that would also be beneficial to manufacturers who deal with multiple countries. Apart from sharing these guidelines, experts from EU OMCLs shared their experiences with COVID-19 vaccines in online meetings with the WHO-NNB Network and also with other global/regional networks (such as the Asian Pacific Economic Cooperation (APEC)). EDQM also shared the EU OCABR network approach and successes with the Regulatory Advisory Group /COVAX to promote reliance approaches.

Sharing product specific guidelines also provides transparency to countries that receive vaccines with an OCABR certificate. For all batches that are provided with an OCABR certificate it is clear that all tests in the specific guidelines have been carried out by an OMCL and that both these test results and the protocol review were found compliant. For vaccines supplied through COVAX, release by a national control laboratory is a requirement and countries accepting these vaccines were strongly encouraged to accept existing release certificates and not retest these batches. Therefore, many batches have been supplied to the global market with EU OCABR certificates. This is especially true for batches that were purchased by EU member states, but instead of being used in national vaccination campaigns, were donated to COVAX. It is important to highlight that all vaccines provided with an OCABR certificate comply with the same standard and there is no difference between vaccines intended for EU and non-EU markets, indeed OMCLs are usually not aware of the final destination of the vaccine batches being tested.

## Lessons learned and moving forward

A number of lessons have been learned in the OCABR Network through managing the COVID-19 crisis. One of the key lessons is that it is possible to accelerate the OCABR process using emergency measures, however taking everything in balance, under normal conditions, it is not necessary to employ all of the emergency measures. During the pandemic, the key challenge was the large volume of batches and short time to prepare.

Several of the elements used during the emergency were already established as part of the normal system but proved to be particularly useful and will be encouraged further, for example:

- Having competent, experienced OMCLs with existing hands-on know how on vaccine testing allowed rapid reactivity and adaptation.
- Designating more than one OMCL for release of a given product, especially for high volume products, was important to ensure business continuity and to spread risk.
- Having common meetings between the manufacturer and all of the OMCLs involved in OCABR for the same product was beneficial both with respect to economising time but also to allow better mutual support and technical exchange between all parties.
- Close contact with the manufacturers regarding the planning of shipment and receipt of samples allowed the optimisation of test schedules to cover multiple batches in the same test run as far as possible, to economise resources and speed up testing.
- Parallel testing is employed widely in normal conditions but was critical to the rapid release of the COVID-19 vaccines.
- The strategic choice of tests for OCABR, with a focus on the added value for independent review, continued to be important and the ability to focus from the beginning on in vitro tests as developed by the manufacturers, instead of animals tests, was a clear advantage.
- The network system with recognition of certificates and testing a given batch in only one OMCL within the EU/EEA has always been an advantage. The COVID-19 crisis illustrated clearly the benefits of this and highlighted the need to maintain and develop the networking aspect.
- The central role of EDQM in coordination of the network activity allowed good points of contact for interaction with manufacturers and external partners and between OMCLs.
- Increased use of video meetings to bring all the actors together on short notice were instrumental to problem solving and rapid decision making at the network level.

In the event of future emergencies, the following lessons should also be considered:

- Interacting with organisations at the focal point of the activity, e.g., EU Commission, EMA, CEPI/COVAX, with clear points of contact and open communication channels was essential.
- Rapid and early exchange with the involved manufacturers was critical. Early access to testing plans, method details and samples to test during the method transfer validation was essential to stay in step with the accelerated authorisation process.
- Facilitating reliance on OCABR certificates outside the EU benefited from the activities of CEPI/COVAX with their members, which helped to improve access to vaccine doses around the world.
- Wide dissemination of OCABR guidelines and documents via partners such as CEPI/COVAX and WHO helped to promote reliance on OCABR certificates worldwide.
- Targeted contact with ‘newcomer’ manufacturers to explain the EU system was beneficial and was facilitated through contacts with EDQM, EMA and CEPI/COVAX; A slow point in the interaction with some COVID-19 manufacturers was making firm arrangements with OMCLs to carry out OCABR (especially manufacturers outside the normal EU vaccine environment). Some were forthcoming with necessary details regarding their products and testing requirements whereas others were very reluctant to share information.

Finally, during the pandemic OMCLs implemented analytical procedures from manufacturers earlier than usual i.e., once the proof of concept was defined but before final validation by the manufacturer. This was necessary to allow the tight deadlines to be met but it increased the probability of late stage adaptations and increased the burden on the OMCL. The urgency of the situation warranted this approach however under normal conditions ideally the validation of the analytical procedures by the company should be completed before transfer to the OMCL and the intention and schedule for the marketing authorisation application should be firm.

## Conclusion

The global health emergency generated by the COVID-19 pandemic has required an incredible mobilisation of resources on many levels to ensure public health. The development and regulatory approval of vaccines against COVID-19 was an important part of this mobilisation. The unprecedented speed of this development and regulatory approval presented many challenges, in particular at the beginning when there were many questions and few concrete answers.

In parallel to the vaccine development, the tools for independent control of these vaccines were also put in place. In the EU the challenges for independent control, including testing, by OCABR were recognised but the importance of the exercise of providing batches of an assured quality to the public was considered paramount to public health and public confidence.

In implementing OCABR for vaccines against COVID-19, the EU OCABR network has used its strengths, and developed new tools and approaches including an enhanced communication within the network and with all relevant stakeholders. The established principles of mutual recognition of batches in the EU/EEA via the OCABR certificate, the use of parallel testing to reduce the time needed for independent release and the work sharing and cooperation between the network members are good examples.

The strategy taken by the network was to focus on science and a risk based approach with selection of key tests based on scientific criteria and an evaluation of needs related to the complex supply situation involving multiple production sites and new players in vaccine manufacturing. New tools included the higher reliance on digital communication and electronic submission of protocols, as well as the implementation of new methods for in vitro testing, development of new guidelines, and the exceptional advance publication of guidelines, with only the list of tests, to promote transparency at an early stage. EU OMCLs have also channelled their resources into the WHO-NBB thus sharing their experience with a wider group of NCLs globally and promoting reliance. These OMCL activities demonstrate reliance/recognition, work sharing between the OMCLs, use of approaches based on science and a risk assessment, and an increased use of digital tools. These are key elements noted by vaccine industry to achieve accelerated supply of vaccines to the market^27,28^.

With the independent control of billions of doses of COVID-19 vaccines, used in Europe, and around the world either via CEPI or directly by other authorities, EU OMCLs have made an important contribution to the fight against the pandemic.

## Data Availability

The authors declare that the data supporting the conclusions of this Perspective are published within the paper.
